# Biological effects of gamma-ray radiation on tulip (*Tulipa gesneriana* L.)

**DOI:** 10.7717/peerj.12792

**Published:** 2022-01-19

**Authors:** Yirui Li, Li Chen, Xiaodie Zhan, Liang Liu, Feihong Feng, Zihua Guo, Dan Wang, Hao Chen

**Affiliations:** 1 Breeding Platform of Sichuan Radiation Mutagenesis Technology, Chengdu, China; 2 National Co-innovation Center for Nuclear Waste Disposal and Environmental Safety, Southwest University of Science and Technology, Mianyang, China; 3 College of Life Science and Engineering, Southwest University of Science and Technology, Mianyang, China

**Keywords:** Tulip mutation breeding, Radiation-biological effects, Micromorphology, Chromosomal division behavior, Phenotypic variation, ISSR analysis

## Abstract

Tulip, being an important ornamental plant, generally requires lengthy and laborious procedures to develop new varieties using traditional breeding methods requires. But ionizing radiation potentially accelerates the breeding process of ornamental plant species. The biological effects of γ-ray irradiation on tulip, therefore, were investigated through establishing an irradiation-mediated mutation breeding protocol to accelerate its breeding process. ISSR-PCR molecular marker technique was further used to identify the mutants of phenotypic variation plants. This study showed that low irradiation doses (5 Gy) stimulated bulb germination to improve the survival rate of tulip, while high irradiation doses (20 to 100 Gy) significantly (*P* < 0.05) inhibited its seed germination and growth, and decreased the flowering rate, petal number, flower stem length and flower diameter. More than 40 Gy significantly (*P* < 0.05) decreased the total chlorophyll content and increased the malondialdehyde (MDA) content in tulips. Interestingly, three types of both stigma variations and flower pattern variations, and four types of flower colour variations were observed. With increasing the irradiation dose from 5 to 100 Gy, the anthocyanin and flavonoid contents continuously decreased. Scanning electron microscopy (SEM) analysis evidenced that high irradiation doses altered the micromorphology of leaf stomata. Microscopic observations of tulip root apical mitosis further showed the abnormal chromosomal division behaviour occurring at different mitotic phases under irradiation treatment (80 Gy). Increasing the irradiation dose from 20 to 100 Gy enhanced the micronucleus rate. Moreover, the suspected genetic variation in tulips was evaluated by inter-simple sequence repeat (ISSR) analysis, and the percentage of polymorphic bands was 68%. Finally, this study concludes that that 80 Gy may be an appropriate radiation does to better enhance the efficiency of mutagenic breeds in tulip plants. Using γ-ray irradiation, therefore, is expected to offer a theoretical basis for mutation breeding in tulips.

## Introduction

Tulip (*Tulipa gesneriana* L.), being an herbaceous ornamental plant (*i.e*., family *Liliaceae)*, is cultivated flower plants worldwide, especially in Europe (Pourkhaloee et al., 2018). Considering a high commercial value due to its various colour, long flowering period, and pleasing fragrance, it has been regarded as the national flower by many countries, such as Turkey, Holland, and Iran (Mu et al., 2020). Among the 10 ten cut flowers sold in several global markets, its demand in the domestic market is also growing with the rapid development of the economy in China. The high popularity of tulips has led to the need to cultivate new varieties with new shapes and colours, which is also suitable for production. Generating tulip varieties with improved traits, thus, has become a popular research topic.

Several methods are widely applied to enhance seed breeding in ornamental plants, including cross breeding, selective breeding, monadic breeding and polyploid breeding (Kishi-Kaboshi, Aida & Sasaki, 2018; Ahn et al., 2020). However, most of these techniques have some disadvantages, such as a long breeding period and heavy workload (Li et al., 2021). Since it has been an important tool to induce mutations and increase the genetic variability in plant breeding, mutation breeding can effectively solve these drawbacks (Kishi-Kaboshi, Aida & Sasaki, 2018). In recent years, this method has created an increasing number of fine varieties and new germplasm resources in flowering plant breeding (Hernández-Muñoz et al., 2019). Gamma-ray irradiation, a typical type of irradiation technology, induces both direct and indirect biological damage on plants by changing DNA structure, thereby promoting the production of new varieties of ornamental plants in a short cycle (Asare et al., 2017; Yamaguchi, 2018). In mutation breeding, both germination percentage and survival rate are the criteria for determining induced-mutations in plants, as these criteria are highly relevant to the generation of mutants. Moreover, the phenotypes of these mutants include flowers with various colours, shapes, and sizes (Noman et al., 2017). It has been observed that gamma-ray irradiation in chrysanthemum (*Dendranthema grandiflorum*) significantly inhibits plant germination and growth, and changing flower colour (Kim et al., 2016). Recently, Yue & Ruter (2020) also experimentally confirmed that gamma-ray irradiation is effective in inducing genomic variations in three Pavonia species, resulting in the changes of germination percentage, flower size, leaf area, leaf chlorophyll, and plant height.

Irradiation also causes the physiological, cytological, and genetic changes in cells and tissues, which alters plant morphology. Fan et al. (2014) reported that γ-irradiation induced a substantial increase in malondialdehyde (MDA) content, but reduced the total chlorophyll content in *Zizania latifolia* plants, suggesting a potential mechanism of plant growth inhibition under irradiation stress. In addition, it has been demonstrated that DNA fragmentation and cell death may inhibiter plant growth and development (Yamaguchi, 2018; Yue & Ruter, 2020). Yamaguchi et al. (2008) found that gamma-ray irradiation, even with low doses (15 Gy), reduced the nuclear DNA content in chrysanthemum, suggesting that irradiation efficiently induced mutations in the plants. Indeed, gamma-ray irradiation induces a sufficient frequency of mutations in plants. Using ISSR markers to further analyze the mutants may offer a useful molecular marker for the detection of mutants in the plant, which provides some valuable information for future breeding of ornamental plants (Wu et al., 2020). Therefore, studying the physiological, cytological, and genetic effects of gamma-ray irradiation on tulip (*Tulipa gesneriana* L.) is of great importance for breeding new tulip varieties and improving the quality of flowering plants.

Here, we hypothesized that gamma irradiation had the potential to accelerate the breeding process of tulips. Thus, this study aimed to indicate the biological effects of irradiation with gamma rays on tulips through determining the optimal dosage for mutation breeding and the potential fertile mutants. The objectives were to (1) investigate the effects of gamma-ray irradiation on plant morphological parameters, (2) assess the effects of gamma-ray irradiation on the physio-biochemical parameters of tulips, (3) explore the alteration of the ultrastructure of tulip leaves and root apical mitosis caused by γ-ray irradiation, and (4) perform ISSR marker analysis in γ-ray-treated tulips.

## Materials and Methods

### Plant material and irradiation

This study selected flowering and healthy tulip bulbs. ‘Queen Days’ is a double- and late-flowering tulip variety being imported from Holland. ^60^Co-γ was used as the radiation source for tulips in these experiments. The tulip bulbs (720) were irradiated by γ-ray irradiation at the Sichuan Institute of Atomic Energy (Chengdu, Sichuan Province, China) at dosages of 0 (CK, control), 5, 10, 20, 40, 60, 80, and 100 Gy at a dose rate of 2.57 Gy·min^−1^. Triplicate repetitions per treatment were conducted, and 30 bulbs were used in each repetition.

### Plant cultivation

All treatments were performed in a greenhouse (10–25 °C) at the Laboratory of Nuclear Waste and Environmental Security in Mianyang. Irradiated bulbs were planted into flowerpots (L × W × H = 49 × 20 × 18 cm) in the greenhouse, with a total of 30 bulbs per flowerpot (January 20, 2018). The soil was composed of special plant cultivated soil, fermentative organic substrate, leaf mould, and vermiculite, and the volume ratio of the above four cultivated soils is 5:2:2:1. The soil moisture content was regularly adjusted to approximately 70% of the field moisture capacity in the experiment. All treatments were arranged according to a completely randomized design (triplicate).

### Plant growth and development parameters

Vegetative and reproductive growth period data, including germination rate, survival rate, flower shape, and colour change, were recorded from the beginning of tulip planting. The germination percentage of plants from each treatment was quantified 15 day (d) after planting when the shoot tip of the bulb was unearthed. At 45 d after planting, the survival rate was quantified, and plants with yellow stems or yellow leaves were considered dead. After 63 d of cultivation, the flowering rate was measured. Additionally, 0.25 g fresh leaves of tulip were extracted with 80% acetone aqueous solution to determine the contents of both chlorophyll a and b using UV spectrophotometer at 665 and 649 nm (Li et al., 2021). The MDA content in these samples was determined with the thiobarbituric acid reaction using the procedure developed by Chen et al. (2020).

### Apical cytology

Three samples were randomly selected for hydroponics. At 9 am (the root tip growth to 1–2 cm), root tip samples were collected and pre-treated with 5 mL of polythene pipe at 4 °C for 24 h. Then, the pre-treatment solution was decanted, Carnoy’s fixative was added Samples after pre-treatment was added Carnoy’s fixative (anhydrous ethanol: glacial acetic acid = 3:1 by volume) at 4 °C for 24 h. Finally, the samples with fixing solution was discarded, washed with deionized water with 70% ethanol for three times, and placed in the refrigerator at 4 °C.

For testing, root tips were removed, washed, and added by 5 mL hydrochloric acid. Pre-treated samples were then heated in a water bath at 60 °C for 6 min and dissociated at room temperature for 4 min. Then, 0.5% carmine acetate staining was performed for specimen preparation. Special morphological changes during the mitotic period were observed. Three visual fields were observed at the root tip for three repeats to count the number of micronuclei and cells.

### Scanning electron microscopy (SEM) analysis

Three plants were randomly selected from each treatment. Samples were randomly collected in the middle of the blade, freeze-dried for 12 h and then sprayed with gold. Scanning electron microscopy (SEM) images were collected using a Zeiss EVO 18 SEM at 20 kV.

### HPLC analysis of anthocyanins and flavonols

Anthocyanins and flavanols were extracted as described above for fresh petal (both ends of the petals were removed) using a methanol:H_2_O:HCl:formic acid:trifluoroacetic acid (70:27:2:1, v/v/v/v) solvent. Liquid extracts were collected to place at 4 °C in the dark for 24 h, and then filtered through a 0.22 μm microporous filter before use (Yuan et al., 2014). Anthocyanins and flavanols were analyzed using an U1tiMate 3000 DGLC HPLC-PAD system. Samples (10 μL) were loaded onto a Zorbax SB-C18 (4.6 × 250 mm, 5 μm) column, stood at 0.8 mL·min^−1^ and separated at 35 °C. Mobile liquid phase A was a 1% aqueous solution of formic acid, and liquid B was acetonitrile. The gradient elution procedure was as follows: 0% A, 10% B, 0 min; 38% A, 62% B, 13 min; 10% A, 10% B, 15 min; 10% A, 90% B, 17 min; 90% A, 10% B, 19 min; and 90% A, 10% B, 21 min. The wavelengths used for anthocyanin, flavone and flavanol detection were 520 and 350 nm, respectively. Anthocyanins and flavonoids were quantified separately using cyanidin-3-O-glucoside (Maclin, Analytical Reference) and rutin (Chengdu Manster Biotechnology Co., Ltd.:99.95% HPLC) standards.

### ISSR-PCR amplification and analysis

Untreated tulip (CK) and mutant plants were collected to extract cDNA using CTAB method. One hundred common PCR primers for primer screening were synthesized by Bioengineering (Shanghai) Co., Ltd. Selected primers were tested for polymorphisms with cDNA extracted from tulips. Reactions were performed in triplicate and optimized as follows: 1 μL of diluted cDNA, 1 μL of 0.2 μmol·L^−1^ primer, 0.1 μL of Taq enzyme, 1.5 μL of dNTPs, 1 μL of MgCl_2_, 2.5 μL of 10× PCR Buffer, and 17.91 μL of ddH_2_O to a total volume of 25 μL. The PCR amplification reaction conditions were as follows: preincubation at 94 °C for 5 min, then 40 cycles of 94 °C for 30 s, 58 °C for 30 s, and extension at 72 °C for 1 min, and a final extension at 72 °C for 7 min with reaction termination at 12 °C. The ISSR-PCR products were evaluated on 1.5% agarose gels with 1 μL of GoldView I type nucleic acid dye. Then, 5 μL of PCR product was mixed with 2 μL of loading buffer. The electrophoresis apparatus contained 1× TAE buffer was run at 120 V for 45 min to detect bands.

### Statistical analysis

To calculate the germination percentage, survival rate and flowering plant rate in each treatment as follows:

Germination percentage = (number of budding bulbs/total bulbs) × 100%;

Survival rate = (number of surviving plants/total number of bulbs) × 100%;

Flowering plant rate = (number of flowering plants/total number of surviving plants) × 100%;

Plants with the same growth potential were selected for each treatment to construct the regression equation of leaf area. This study was to take the distance from the blade tip to the petiole as the length (L) and the widest part of the blade as the width (W). The straight edge was estimated to be 0.01 cm. The actual leaf area (s) was measured using WYD-500a microelectronic area measurement. The regression equation of leaf area was constructed as *y* = 0.74x + 4.45 (R^2^ = 0.98) to calculate the total leaf area of each treatment.

In the study, all data were represented as means ± standard deviations (triplicate) using the analysis method from Tchokponhoue, N’Danikou & Achigan-Dako (2019), to determine the least-significant difference (LSD) between different treatments (*P* < 0.05) using SPSS software (22.0). All figures were drawn by Origin 2020a.

## Results

### Effects of different irradiation doses on the plant growth parameters of tulips

#### Bulb germination and survival of tulips

**Figure 1:**
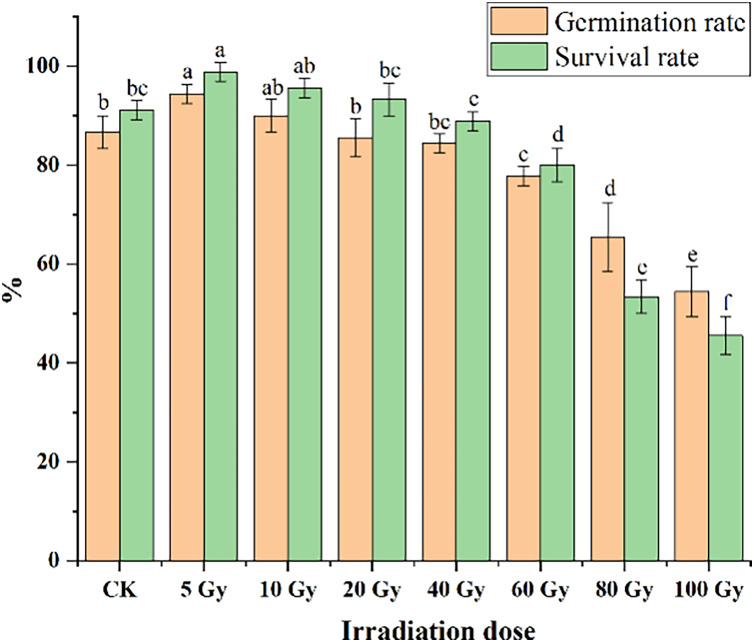
Influences of different irradiation doses on germination rates (15 d) and survival rates (45 d) of tulips. Note: all data were represented as means ± standard deviations (triplicate). Lowercase letters represent statistically significant differences at *P* < 0.05. Error bars indicate the standard error of the mean.

Different irradiation doses caused a significant difference in both the germination and survival rates of tulips (Fig. 1). Low irradiation doses (5 and 10 Gy) increased the germination and survival rate of tulip bulbs, while higher irradiation doses (60 and 80 Gy) exerted significant negative effects (*P* < 0.05). The maximum germination (94.44%) and survival rates (98.89%) were observed with the 5 Gy treatment, and significantly higher than that in CK (86.67%) (*P* < 0.05). With increasing irradiation doses, both germination and survival rates decreased gradually. Under 10 Gy treatment, the germination percentage was slightly higher than that in CK. Moreover, the germination rates of 20 and 40 Gy were slightly lower than that in CK, but not significantly. The survival rate decreased from 98.89% to 93.33% with increasing radiation doses from 5 to 20 Gy, but their survival rates were still higher than that in CK.

#### Morphological and flower-related traits of tulips

**Figure 2:**
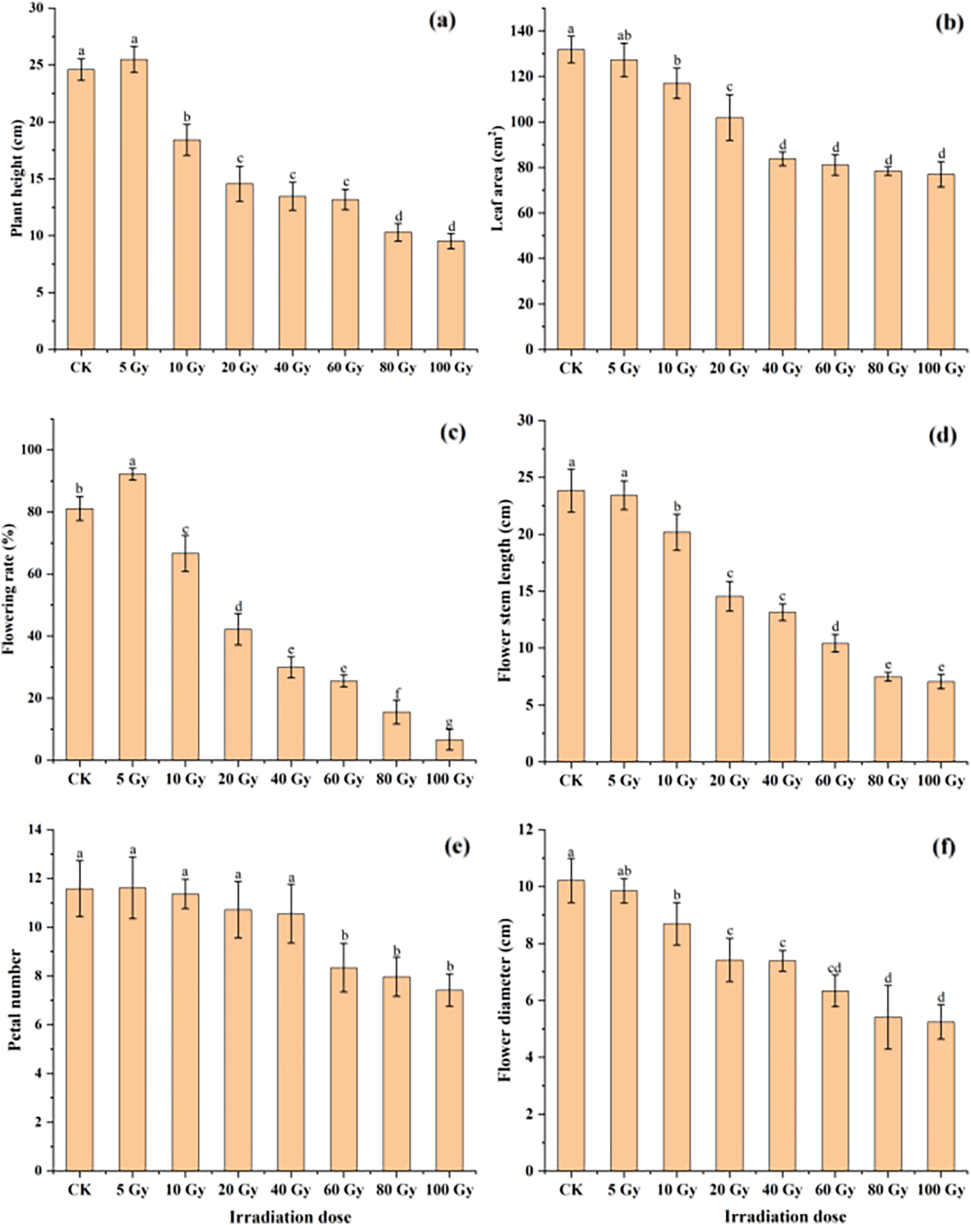
Influences of different irradiation doses of γ-rays on plant height (A), leaf area (B), flowering rate (C), flower stem length (D), petal number (E) and flower diameter (F) of treated tulips. Note: all data were represented as means ± standard deviations (triplicate). Lowercase letters represent statistically significant differences at *P* < 0.05. Error bars indicate the standard error of the mean.

The effect of different γ-ray irradiation doses on the morphological and flower-related traits of tulips are given in Fig. 2. Under 5 Gy treatment, plant height slightly increased. Yet the plant height significantly decreased with increasing irradiation doses from 10 Gy to 100 Gy. For the leaf area, all γ-ray irradiation doses resulted in a decrease compared with the CK (Fig. 2B). Moreover, a significant inhibition (*P* < 0.05) in the leaf area was observed under the range of the irradiation dose (10–100 Gy).

Similarly, the flowering rate and petal number first increased in the 5 Gy treatment and then decreased with increasing irradiation doses from 5 to 100 Gy (Fig. 2C and 2E). Moreover, both the flowering stem length and diameter continuously decreased with increasing γ-ray irradiation doses (Fig. 2D and 2F). The flowering rate, petal number, flowering stem length and flowering diameter were 6.67%, 7.42, 7.06 cm and 5.24 cm in the 100 Gy irradiation treatment, which were decreased by 91.78%, 35.98%, 70.37% and 48.68%, respectively, compared to those in the CK.

### Phenotypic analysis of flowers

#### Analysis of stigma variation

**Figure 3:**
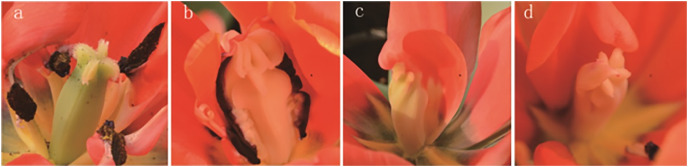
Stigma variation of treated tulips. Note: (A) Control. (B) The top is lantern-shaped and connected to the petals. (C) Connected to the petals. (D) Apical claw.

The results of the phenotypic analysis after irradiation are displayed in Fig. 3. Three shapes of tulip stigmas, three kinds of colour mutants, and four types of florescence were observed. As shown in Fig. 3A, the stigmas of untreated controls (top of stigma) were cross shaped, and the ovaries (bottom of stigma) were four-corner rhombohedrons. In the mutants, the top of the stigma was lantern-shaped, while the ovary, stamens and petals were adhered and displayed some white bulges (Fig. 3B). In some plants, one side of the stigma was connected to the petals, while the ovary edge disappeared, and the top of the structure was abnormal (Fig. 3C). The third type of stigma was curled at the top, and the petals had degenerated (Fig. 3D).

#### Tulip colour and pattern changes

**Figure 4:**
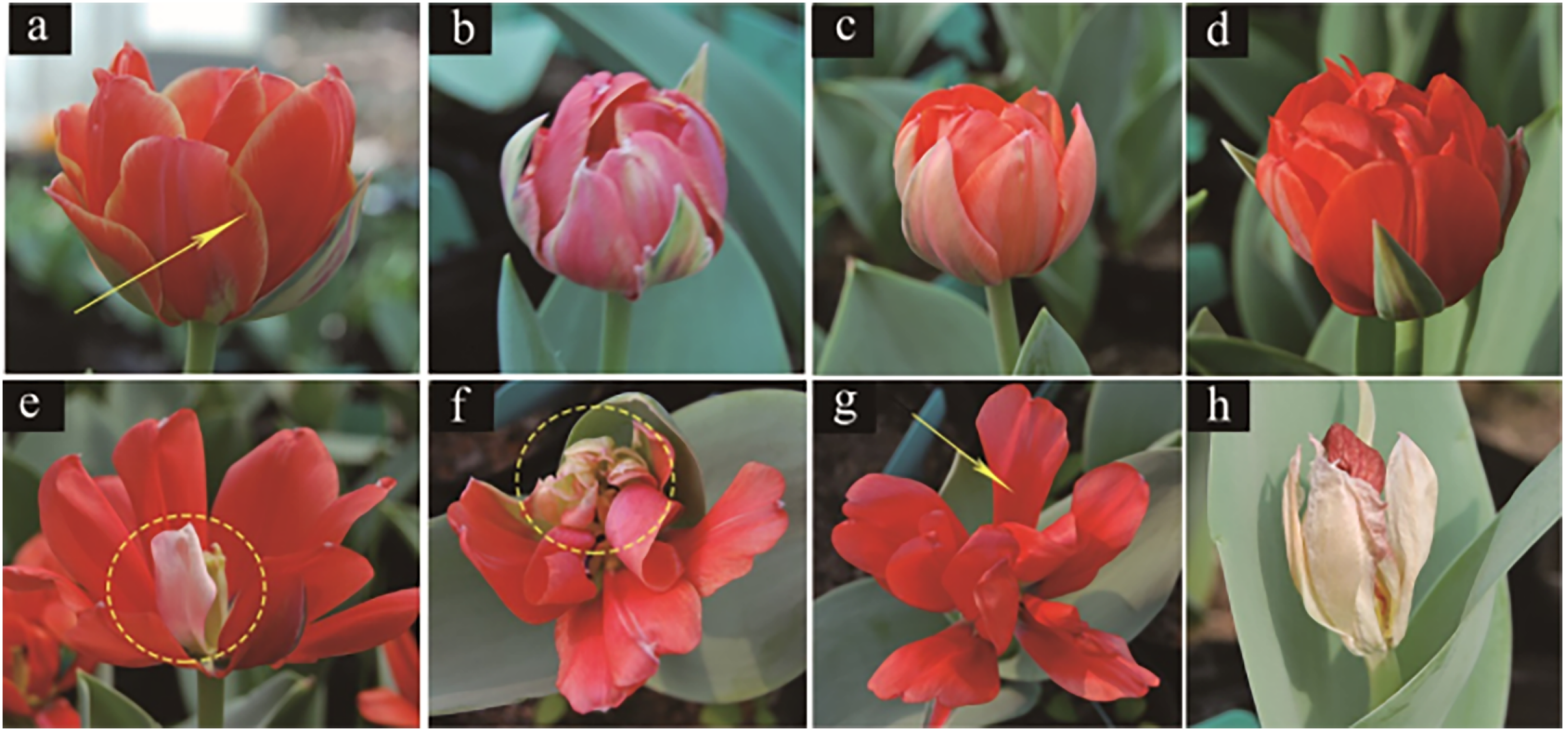
Flower colour and petal variation in treated tulips. (A) Control. (B) Pink plant. (C) Orange plant. (D) Red plant. (E) White petals. (F) Petal degeneration. (G) Petal narrowing. (H) The flowers are not blooming and are colourless.

The variation in both the colour and pattern of tulip flowers under irradiation treatments are shown in Fig. 4. For the colour, untreated tulip petals were orange inlaid with yellow at the edge, and the calyx was atrovirens, ivory and pink (Fig. 4A). The treated tulip petals turned pink, which was mixed with white. Moreover, the petals shrank and the yellow colour disappeared from the edge (Fig. 4B) or turned pure orange; the colour of the calyx was the same as that of the petals and the calyx was extremely petal-like (Fig. 4C). Alternatively, some flowers were red (Fig. 4D), with part of the petals being white (Fig. 4E). With respect to pattern, tulip petals in the control group were obovate, large and closely arranged (Fig. 4A). The treated tulip petals were degraded and light (Fig. 4E) or severely degraded on one side of the petals, causing the pattern to be “fan-shaped” (Fig. 4F). The petals of some tulips were narrowed and became long ovate, with adjacent petals not tightly connected (Fig. 4G) or all petals degenerated; flowers were the same shape as buds, with most petals thin, wrinkled, and white, with a degenerated and disappeared calyx (Fig. 4H).

#### Effects of different radiation doses on the total chlorophyll and MDA contents of tulips

**Figure 5:**
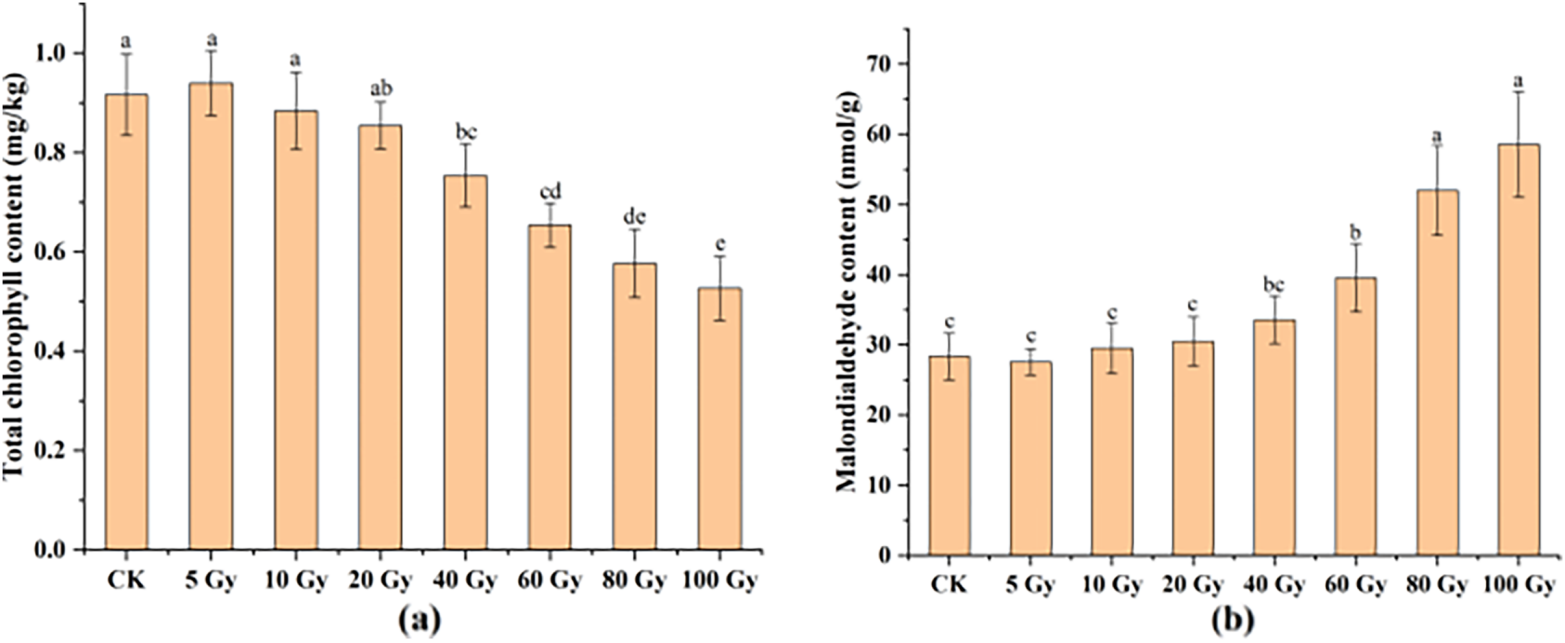
Total chlorophyll content (A) and MDA content (B) of tulip leaves. All data were represented as means ± standard deviations (triplicate). Lowercase letters represent statistically significant differences at *P* < 0.05. Error bars indicate the standard error of the mean.

This study examined the chlorophyll and MDA contents in tulip leaves (Fig. 5). The maximum total chlorophyll content of tulip leaves was 0.94 mg kg^−1^ under the 100 Gy treatment, which was slightly higher than that of the CK. Total chlorophyll content of the tulip leaves continuously decreased under the irradiation dose above 10 Gy (Fig. 5A). Conversely, the MDA contents of the tulip leaves increased with increasing γ-ray irradiation dose (Fig. 5B). Moreover, there was no significant differences between the CK and either 5, 10, 20, or 40 Gy treatments. The MDA content was 58.56 nmol·kg^−1^ in the 100 Gy irradiation treatment, which was 106.56% higher than that in the CK. These results indicate that γ-ray irradiation at high levels caused severe stress to the plants.

#### Preliminary analysis of pigment content

Anthocyanin and flavonoid contents in tulip petals at each radiation dose were determined (Figs. S1–S2). The colour of the tulips showed an obvious change after irradiation. The interference caused by the extraction solution was excluded, and the peak at 2.5~5 min was not counted when calculating the content (Fig. S1). HPLC chromatograms of tulips were evaluated at 520 nm, but four anthocyanin components were detected. In addition, more than 10 flavonoids were detected at 350 nm. During detection, some plants with obvious abnormal peaks of anthocyanins and flavonoids were selected (Fig. S2).

**Figure 6:**
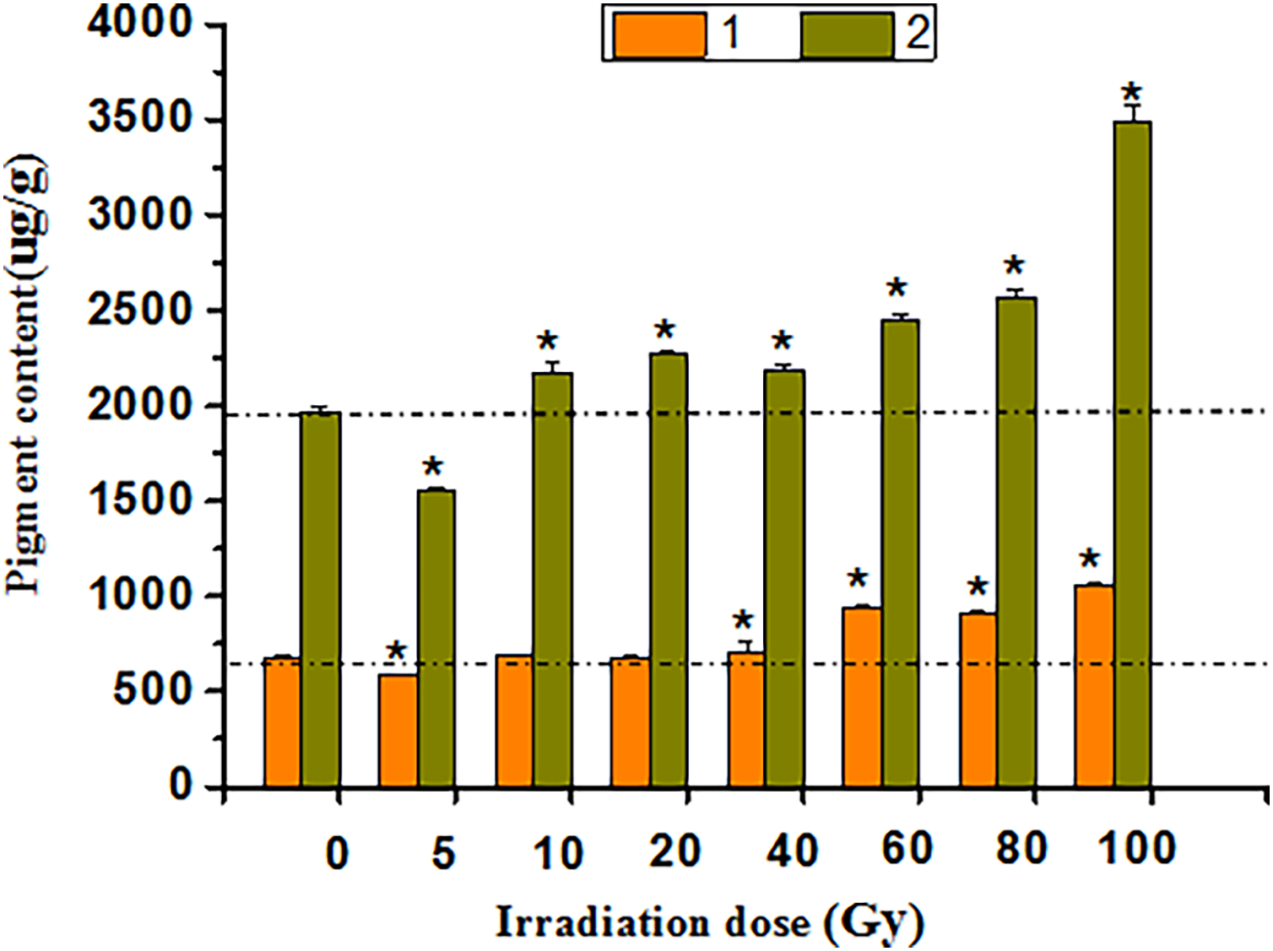
Chart of total anthocyanins and flavones in treated tulips. An asterisk (*) indicates significant differences among treatments, *P* < 0.05. Note: “1” indicates the anthocyanin content of petals at each irradiation dose, and “2” indicates the flavonoid content of petals at each irradiation dose. All data were represented as means ± standard deviations (triplicate). Error bars indicate the standard error of the mean.

The HPLC study showed that anthocyanin and flavonoid contents were positively correlated with irradiation doses of 5–100 Gy (Fig. 6). After 40 to 100 Gy irradiation, the anthocyanin content was significantly higher than that in the CK (*P* < 0.05), whereas the maximum value was 1,055.94 μg·g^−1^, which was 55.30% higher than that in the CK. Flavonoids increased significantly from 10 to 100 Gy (*P* < 0.05), with a peak of 3,491.53 μg·g^−1^ at 100 Gy, which was 77.21% above that of the CK.

### Mutagenic effect of γ-ray irradiation on tulip

#### Scanning electron microscopy detection in leaf

**Figure 7:**
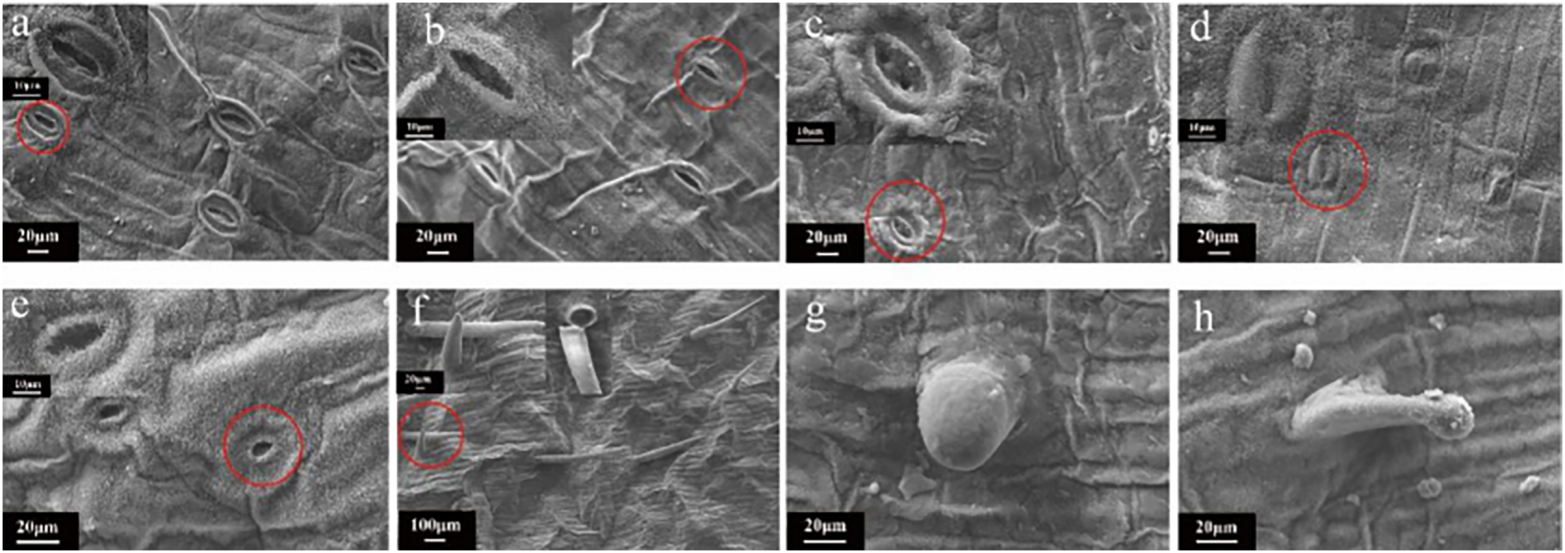
SEM images of the morphology of the trichome on the surface of the tulip leaves under different doses of irradiation. CK (A); 5 Gy (B); 20 Gy (C); 40 Gy (D); 80 Gy (E); 40 Gy (F); 60 Gy (G); and 100 Gy (H).

Scanning electron microscopy (SEM) imaging revealed that the morphology of leaf stomata changed after irradiation treatment (Fig. 7). This morphology was different than that typically observed when the leaf stomata were evenly distributed and oval. Moreover, there was no adhesion or closure of the stomata (Fig. 7A). Then, samples were randomly selected from each radiation dose for SEM observation, showing that the contour of the stomata was blurry or even vanished at 5 Gy (Fig. 7B). Intracellular adhesions of stomata left only tiny pores at 20 Gy (Fig. 7C). With increasing irradiation dose, the stomatal morphology was seriously damaged with a blurred edge and the loss of its opening and closing function (Fig. 7D). Some stomata were hollow (Fig. 7E). Figs. 7F–7H showed SEM images of the morphology of the trichome on the surface of the tulip leaves, responding irradiation at high doses (60 to 100 Gy). The surface of the trichome was smooth, hollow inside, and shaped as a tip or cone (Fig. 7F) or ball (Fig. 7H).

#### Microscopic observations of tulip root apical mitosis

**Figure 8:**
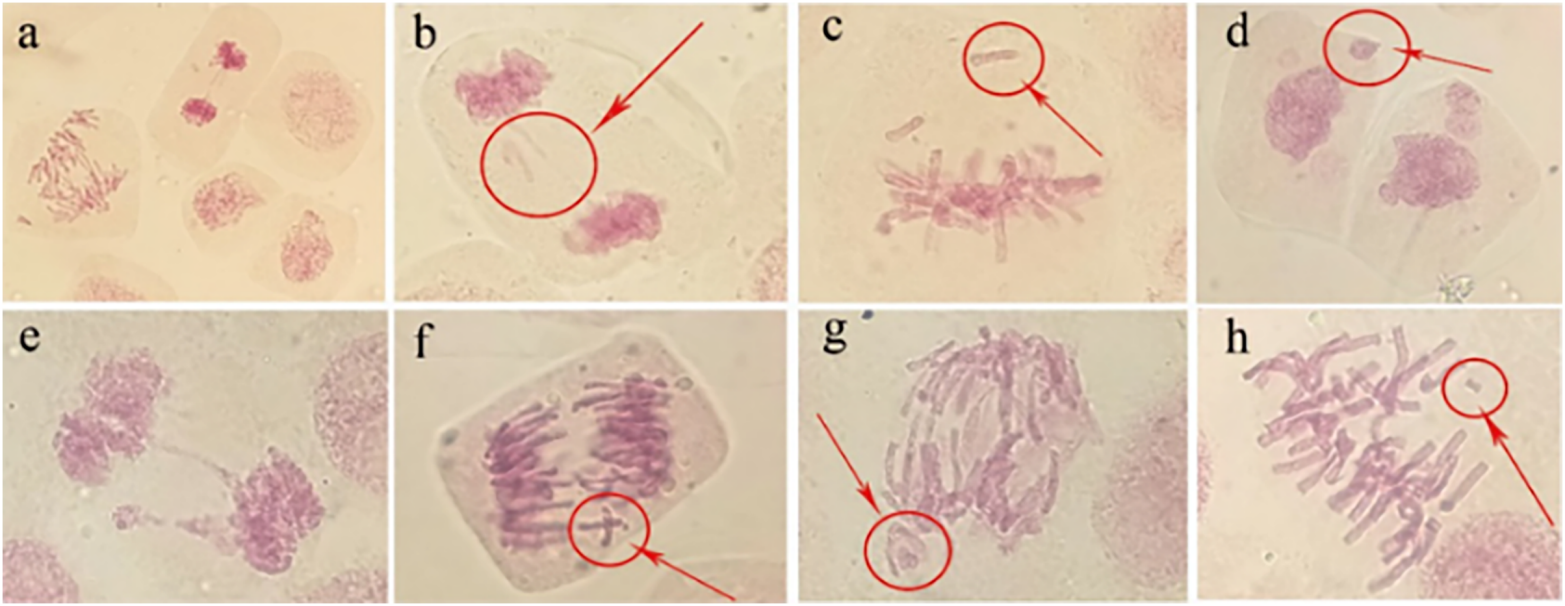
Observation of the mitotic behaviour of tulip root tips under irradiation treatment. Observation of the mitotic behaviour of tulip root tips under irradiation treatment. Mitotic cell morphology at different stages in the root tip (A); chromosome bridge and laggard chromosome (B); unpaired chromosome (C); micronucleus (D); chromosome bridge and micronucleus formation (E); centromeric undivided chromosome (F); chromosome moving forward toward both poles (G); and broken chromosome fragment (H).

Abnormal chromosomal division behaviour occurring at different mitotic periods under irradiation treatments (80 Gy) was observed under a microscope (Fig. 8A). During metaphase of chromosome division, when chromosomes were arranged on equatorial plates, free or unpaired chromosomes remained at the poles (Fig. 8C), or broken fragments of chromosomes were scattered (Fig. 8H). During mid-anaphase, centromere division was not complete, and there were unseparated sister chromosomes on the equatorial plate (Fig. 8F). Some of the free chromosomes bound to micronuclei (Fig. 8G). The telophase chromosome bridges were more severe (Figs. 8B and 8E), and there were free chromosomes (Fig. 8B) or complete micronuclei (Fig. 8E). At the end of mitosis, the chromosomal fragments resulting from radiation damage formed micronuclei, which were free around the nucleus (Fig. 8D).

#### Micronucleus frequency in tulip root tips

**Figure 9:**
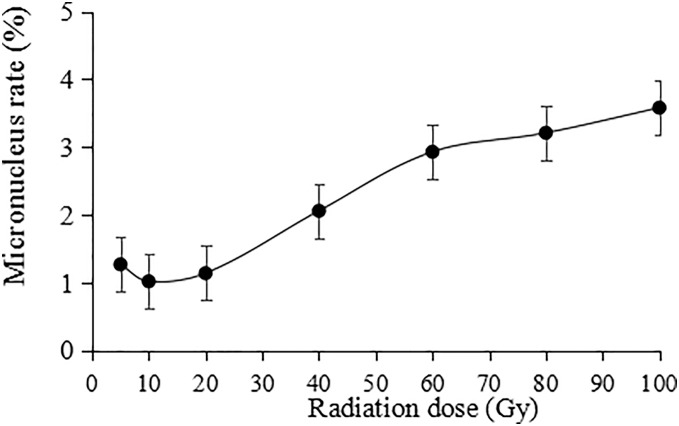
Statistics on the micronucleus rate in tulip root tips under irradiation treatments. All data were represented as means ± standard deviations (triplicate). Error bars indicate the standard error of the mean.

The irradiation dose was divided into two groups: lower dose (5 to 20 Gy) and higher dose (40 to 100 Gy). Statistically, the micronucleus formation rate was positively correlated with the irradiation dose, and the higher the irradiation dose was, the higher the micronucleus formation rate (Fig. 9). Thus, the results indicate that the damaging effect of high-dose irradiation was more significant (*P* < 0.05).

#### Analysis of ISSR polymorphisms in tulip varieties

**Figure 10:**
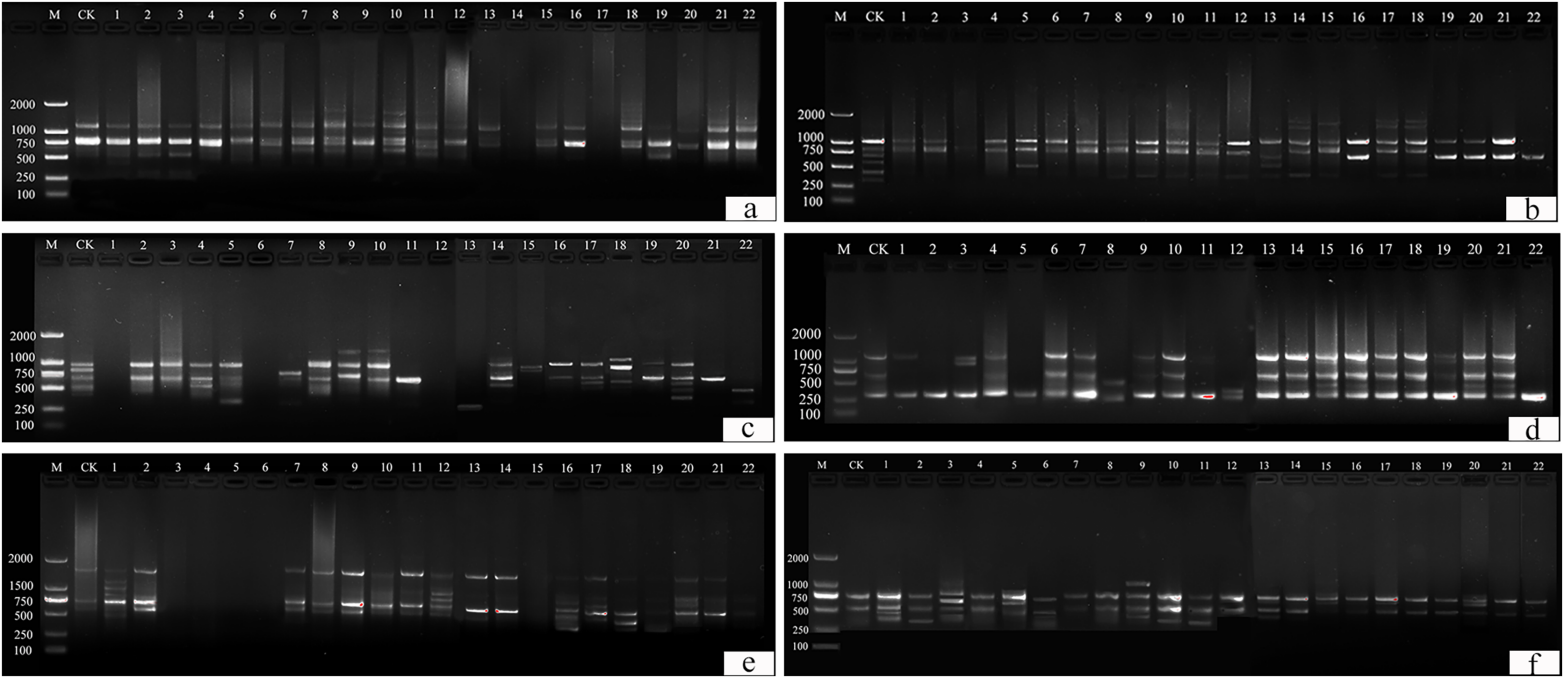
ISSR detection of genetic variation with primer sequences. (A) 825, (B) 830, (C) 846, (D) 847, (E) 855, (F) 856. M is a molecular weight marker; CK is a tulip that was not treated; 1–22 is the plant number.

This study identified 22 plants with suspected variations. Thirteen primers (UBC 817, 825, 826, 829, 830, 847, 848, 849, 855, 856, 859, and 866) were selected from 100 primers for amplification and scoring (Table S1). A ddH_2_O control under the same conditions was used to exclude false positive results. Amplification products ranged from 250 bp to 2,000 bp (Fig. 10).

UBC primers 826, 830, and 849 amplified the largest number of bands (6 bands), and the UBC primer 866 amplified the fewest (1 band). Fifty-two bands were amplified by 12 primers, the number of polymorphic bands of which was 34 and the percentage of polymorphism was 65%. The polymorphic percentages of UBC primers 817, 825, 848, 856, and 866 were the highest, at 100%, and the polymorphic percentages of UBC primers 846 and 859 were the lowest, at 40%.

For UBC primer 825 (Fig. 10A), the entire band was deleted in plants 14 and 17, and additional sites were found in plants 2–3, 6–8, 10–11, 13, 15, and 18, particularly in plants 8, 10, and 11. For UBC primer 830 (Fig. 10B), plants 1–22 all showed partial band deletions, and bands of 1,000–2,000 bp molecular weight were observed in plants 14–15 and 17–18. For UBC primer 847 (Fig. 10D), partial sites were deleted in plants 1–2, 5, 8, 11, and 22, while plants 15–16 and 19–22 showed an increase in size, and plants 3, 8, and 12 showed both an increase and a deletion. For UBC primer 855 (Fig. 10E), plants 3–6 and 15 had no bands, while the others had polymorphisms. UBC primer 856 (Fig. 10F) showed an increase in the number of bands in plants 1–3, 5–6, 9–11, 13, 17, and 20, and a deletion of the bands at 500 bp in plant 15.

## Discussion

### The effects of γ-ray irradiation on the morphological parameters of tulips

The application of mutation breeding technology has increased the production of new varieties of ornamental plants such as chrysanthemum (Patil et al., 2017), rose (Datta, 2018), carnation (Teixeira & Silva, 2014), and tulip (Orlikowska et al., 2018). These varieties have been commercialized (Ibrahim et al., 2018; Yamaguchi, 2018). In mutation breeding, both germination percentage and survival rate highly relate the generation of mutants and are the criteria for determining induced mutations in plants. In this study, the germination percentage of the irradiated seedlings decreased significantly with increasing the irradiation doses from 5 to 100 Gy, suggesting a relative sensitivity of the plants to irradiation. These findings positively agree with germination percentage studies in *Cicer arietinum* L. (Mullainathan, 2016), *Abelmoschus esculentus* L. Moench (Asare et al., 2017), *Sophora davidii* Franch. (Wang et al., 2017), and *Pavonia hastata* (Yue & Ruter, 2020). This reduction in seed germination might be due to the effect of mutagens on meristematic tissues of the seed as well as chromosomal aberrations and interruptions in DNA replication and growth regulators (Wang et al., 2017; Asare et al., 2017). This study also indicated that low germination resulted in low seedling survival as the doses of gamma-ray irradiation increased. The influence of the irradiation on physiological activities and chromosomal damage resulted in a decrease in plant survival (Manju & Gopimony, 2009; Kiong et al., 2008). In addition, a high gamma-ray radiation dose can damage cell membrane integrity and permeability to inhibit the nutrient and water uptake, thus causing interference with plant growth and and physiological activities (Li et al., 2021).

Numerous researchers have reported that gamma rays affect the morphology of treated plants (Yue & Ruter, 2020; Li et al., 2021; Asare et al., 2017). Indeed, this study also found that plant height and flowering rate decreased significantly (*P* < 0.05) with increasing doses of γ-ray irradiation. The reduction in plant height may be attributed to damage cell division and cell elongation processes as a result of mutagenic treatment (Iqbal, 1969). For examples, leaf area, flower stem length, flower diameter, and petal number under irradiation treatments decreased with increasing the irradiation dose (Fig. 2). This is because that irradiation causes DNA breakage in plant cells, further leading to various types of damage to plant cell division and development processes, and plant growth (Amirikhah et al., 2019; Li et al., 2021).

Ionizing irradiation is an effective technique for inducing novel flower colour and shape phenotypes over a short period (Matsumura et al., 2010). After γ-ray irradiation, mutants with floral organ changes, *i.e*., stigma and petals, can be isolated (Kumari & Kumar, 2015). This study also observed the variation in the tulip stigma, showing that the top of the stigma was lantern-shaped and connected to the petals in the mutants. There are two possible reasons for this: first, the meristems follow the central organs during the development of flowers, but the mutations may have caused cell misplacement during the early development of flowers (Kumari & Kumar, 2015). Second, radiation causes differences in homologous genes, resulting in different stigma structures (Scutt & Vandenbussche, 2014; Orlikowska et al., 2018). Moreover, the colours and patterns of treated tulip flowers altered. These phenomena of abnormal flowers can be attributed to the direct effect of radiation on the active and nutrient components of flowers (Yao et al., 2015), and inhibitory effects may be related to auxin and DNA damage after γ-ray exposure (Minisi et al., 2013). HPLC is a common method for the detection of anthocyanin and flavonoid contents in flowers, such as irises (Xu et al., 2018), hibiscus flowers (Grajeda-Iglesias et al., 2017) and strawberry flowers (Xue et al., 2016). The chemical structure of flavonoids can effectively remove the free radicals induced by radiation and reduce the frequency of micronuclei, exerting radiation protection (Weiss & Landauer, 2009). Simultaneously, the control of plant pigments during flowering was obvious and played a specific role in flower development (Muñoz & Munné-Bosch, 2018), which may be a direct reason why mutant tulips cannot blossom. These results suggest that γ-ray radiation, affecting endogenous substances, such as pigments, leads to changes in tulip colour and patterning.

### The effects of γ-ray irradiation on MDA and chlorophyll parameters of tulip plants

Previous studies have reported that gamma rays can affect the biochemistry and physiology of treated plants (Asare et al., 2017; Li et al., 2021), through assessing the effect of γ-ray irradiation on the physio-biochemical parameters of tulip plants, total chlorophyll content and malondialdehyde (MDA) (as an indicator for lipid peroxidation). Chlorophyll contents constitute an important marker to show the effects of stress factors such as γ-ray irradiation (Beyaz, 2020). In this study, the chlorophyll content of the tulip leaves increased at 5 Gy and then decreased with increasing irradiation. It has been reported that low doses of gamma rays (15 krad) significantly increased the total chlorophyll content of tall fescue, but high doses of gamma rays (40 krad) inhibited chlorophyll synthesis in the plants (Amirikhah et al., 2019). In other words, when the levels of radiation increase above the maximum tolerable limit of the plants, the capabilities of the photosynthetic apparatus would be decreased due to the photosystem damagement, thus resulting in a decrease in chlorophyll content (Li et al., 2021).

MDA, being a final decomposition product of lipid peroxidation, is widely used to indirectly determine the physiological condition of the plant response to abiotic stress (Chen et al., 2021, 2022). Our study showed that thers were no significant differences between the control and either 5 Gy- or 40 Gy-treated plants in terms of MDA contents, suggesting that low-level irradiation did not cause severe stress on the plants. The MDA contents were significantly (*P* < 0.05) higher in mutants than that in the CK after 60 to 100 Gy irradiation. This finding is similar to that for 100 and 75 Gy gamma-ray irradiated plants, which showed a significant increase in MDA content of both *Glycine max* (Linn.) Merr. and *Freesia hybrida* (Štajner, Popović & Taški, 2009; Li et al., 2021).

Flavonoids are plant secondary metabolites to help plants cope with the challenges of adverse environment. Previous studies have shown that flavonoids, including anthocyanins and flavonoids, play an active role in plant abiotic stress (Nguyen et al., 2016). Anthocyanins produce colours ranging from orange and red to purple and blue, and flavonoids act as auxiliary pigments for anthocyanin and may produce blue and darker colours (Tanaka & Brugliera, 2013). In particular, anthocyanin produces flower colours from orange to blue based on pH variation, and flavonoids and metal ions enhance the colour by binding to themselves or intermolecular stacking (Tanaka, Sasaki & Ohmiya, 2008). Simultaneously, flavonoids produce the widest range of colours, as colourless compounds coexist within anthocyanins (Gonzalezmanzano et al., 2009). Popescu (2012) found that irradiation relatively varied proportions of different pigments in flowers and significantly altered floral colour, resulting in mutants with various colours, positively corresponding to our study. Interestingly, our results also showed that anthocyanin and flavonoid contents were positively correlated with irradiation doses over the range of 5 to 100 Gy, as reported by El Sherif et al. (2011). A previous study showed that anthocyanin content increased significantly over the range of 40~100 Gy, yellow disappeared from the petal edges, and the flower colours changed from orange to red. Therefore, it was speculated that the higher content of flavonoids in petals was an important reason for the colour change. The increase in flavonoid content in response to γ-ray irradiation resulted in the stacking of anthocyanin and flavonoid molecules, enhancing the flower colour (Gonzalezmanzano et al., 2009). In addition, some studies have shown that the pigment content of flowers was changed by irradiation treatment (Fu et al., 2013). Metal particles and petal epidermal cell shapes could lead to flower variations (Yoshida et al., 2006). Therefore, it can be speculated that the flower pigment was affected by the irradiation treatment, leading to variation in the flower patterns of tulips.

### Alteration of the ultrastructure of tulips caused by γ-ray irradiation

Irradiation does not only cause morphological changes in plants, including thicker leaves, curled leaves and changes in leaf shape, but also affect leaf respiration and photosynthesis (Chen et al., 2016; Li et al., 2021). However, it is still limited on the information regarding the effect of ionizing irradiation on the leaf ultrastructure of ornamental plants. In this study, SEM results revealed that the stomatal morphology changed under irradiation treatment. The contour of the stomata was blurred at the 5 Gy treatment, and intracellular adhesions of stomata left only tiny pores at 20 Gy. In particular, with increasing irradiation doses, stomatal morphology was seriously damaged, with blurred contours and the loss of its opening and closing function. Some stomata were even hollow. These results may be due to obvious changes in the ultrastructure of the leaves (cuticula, epicuticle, palisade tissue, and spongy tissue) after irradiation, especially because the epidermis was distorted and irregular due to tissue disorder (Chen et al., 2016; Rosmala, Khumaida & Sukma, 2016). In addition, the excess energy from irradiation affects late leaf development and deforms the grooming tissue inside the epidermis, mesophyll and leaves, creating a trichome, and leading plants to instinctively close stomata and reduce transpiration rates to maintain growth (Reynolds-Henne et al., 2010). Stomatal closure is a main cause in reducing photosynthesis and affects plant metabolism (Najar et al., 2019), which may be an important factor in plant dysplasia and premature senescence after irradiation treatment.

Similar variation at the cellular level also reflects a change at the biological level (Robson et al., 2015), which has been used as an indicator of the damage degree to plant cells. Microscopic observations of tulip root apical mitosis showed that abnormal chromosomal division behaviour occurred at different mitotic periods. Moreover, irradiated cells may produce two or more mutation types. Some studies have shown that the type of mitotic aberrations, destruction of mitotic devices, and dispersion of chromosomes may all lead to cell decay, while two or more aberrant types are critical levels of aberrations (Kravets et al., 2011). The above chromosome abnormalities may be due to spindle failure and inhibition of tubulin polymerization after irradiation. Moreover, activated oxygen produced by irradiation modifies deoxyribose resulted in single- or double-strand breaks in the DNA and forming chromosomal fragments (Liman, Ciğerci & Öztürk, 2015). Therefore, radiation may cause irreversible damage to the root tip.

### ISSR marker analysis in the γ-ray treated tulips

Polymorphic genetic markers are widely used in plant improvement programs and can identify varieties and parents. They are highly reproducible, polymorphic, informative and easy to use (Chaudhary et al., 2018). ISSR has been widely used to detect changes in DNA, including in *Pimpinella anisum* L. (Giachino, 2019), *Sophora davidii* (Wang et al., 2017), tuberose (Sirohi et al., 2017), *Abelmoschus esculentus* L. Moench (Asare et al., 2017), *Eucalyptus cladocalyx* (Contreras-Soto et al., 2016), *Passiflora* L. (Passifloraceae) (Sousa et al., 2015) and others.

According to ISSR analysis, these results could be due to irradiation causing a deletion or insertion of nucleotides in the DNA sequence, leading to a reading frame shift that caused protein products or faulty transcripts, *i.e*., mutations (Bradshaw, 2016). In addition, the brightness of the amplified ISSR band altered. On the other hand, since direct damage and alterations to DNA are heritable, γ-rays are the primary cause of single-stranded and double-stranded breaks in DNA, and changes in DNA bases (Bradshaw, 2016). Our results have shown that γ-irradiation, being a key to tulip mutation, is an effective way to enlarge the mutation map.

## Conclusions

Overall, this work was conducted to investigate the effects of γ-irradiation with different doses on the biology of tulips. The results of plant growth parameters illustrated that a low irradiation dose (5 Gy) had stimulatory effects on bulb germination, plant growth and flowering, whereas high irradiation doses had more significant inhibitory effects on various plant growth and flowering parameters, including germination rate, survival rate, plant height, leaf area, flowering rate, petal number, flower stem length, and flower diameter. High irradiation doses (60 to 100 Gy) also significantly decreased chlorophyll biosynthesis and enhanced the deleterious effects on the tested plants by increasing the levels of lipid peroxidation (MDA). Simultaneously, γ-ray irradiation was indirectly found to be a main reason for the change in the microscopic morphology of tulip leaves, the pattern and colour of flowers. Additionally, a high irradiation dose (80 Gy) caused abnormal chromosomal division behaviour and significantly increased the micronucleus rate, suggesting that a high irradiation dose induces a biological damage in plant by altering mitotic behaviour. Moreover, ISSR analysis offered a useful molecular marker for the identification of mutant plants. Although this study offers the reference not only for breeding new tulip varieties, but also for mutation research of other ornamental plants, further research is necessary to investigate the molecular mechanisms of mutation breeding by γ-ray irradiation.

## Supplemental Information

10.7717/peerj.12792/supp-1 Supplemental Information 1 Supplementary Figures and Table.

10.7717/peerj.12792/supp-2 Supplemental Information 2 Raw data.

## Additional Information and Declarations

### Data Availability

The following information was supplied regarding data availability: The raw data is available in the Supplemental File.
