# Supplementary material for: Biological effects of gamma-ray radiation on tulip (Tulipa gesneriana L.)
Source: PeerJ. 2022 Jan 19;10:e12792. doi: 10.7717/peerj.12792 (PMC8783560; doi:10.7717/peerj.12792)
Supplement: Supplemental Information 1 [file peerj-10-12792-s001.docx]

**Biological effects of gamma-ray radiation on tulip (*Tulipa gesneriana* L.)**

Yirui Li ^a,b,c,1^, Li Chen ^a,1^, Xiaodie Zhan ^a,b,c^, Liang Liu ^a,b,c^, Feihong Feng ^a,c^, Zihua Guo ^a,c^, Dan Wang ^a,b,c, *^, Hao Chen ^a,b^

^a^ College of Life Science and Engineering, Southwest University of Science and Technology, Mianyang, 621010, Sichuan, PR China;

^b^ Breeding Platform of Sichuan Radiation Mutagenesis Technology ,Chengdu, 621000, Sichuan, PR China;

^c^ National Co-innovation Center for Nuclear Waste Disposal and Environmental Safety, Southwest University of Science and Technology, Sichuan Mianyang 621010, PR China

**Fig. S1.** Determination of anthocyanin HPLC-PAD in petals: a) Extract. b) Control. c) 40 Gy irradiated petals. d) 100 Gy irradiated petals. Anthocyanin extracts from petals of tulips, and the authentic standard cyanidin-3-O-glucoside were subjected to HPLC analysis.

**Fig. S2.** Determination of flavonoids in petals by HPLC-PAD. Note: a) Extract. b) Control. c) 40 Gy irradiated petals. d) 100 Gy irradiated petals. Anthocyanin extracts from petals of tulips, and the authentic standard cyanidin-3-*O*-glucoside were subjected to HPLC analysi.

**Table S1**. ISSR primer sequences of 22 tulip strains and their amplification results

| Primer sequence number | Primer sequence | Total number of bands | Polymorphic bands | Polymorphic percentage |
| --- | --- | --- | --- | --- |
| 817 | CAC ACA CAC ACA CAC AA | 2 | 2 | 100 |
| 825 | ACA CAC ACA CAC ACA CT | 4 | 4 | 100 |
| 826 | ACA CAC ACA CAC ACA CC | 6 | 4 | 67 |
| 829 | TGT GTG TGT GTG TGT GC | 3 | 2 | 67 |
| 830 | TGT GTG TGT GTG TGT GG | 6 | 3 | 50 |
| 846 | CAC ACA CAC ACA CAC ART | 5 | 2 | 40 |
| 847 | CAC ACA CAC ACA CAC ARC | 4 | 3 | 75 |
| 848 | CAC ACA CAC ACA CAC ARG | 3 | 3 | 100 |
| 849 | GTG TGT GTG TGT GTG TYA | 6 | 3 | 50 |
| 855 | ACA CAC ACA CAC ACA CYT | 4 | 2 | 50 |
| 856 | ACA CAC ACA CAC ACA CYA | 3 | 3 | 100 |
| 859 | TGT GTG TGT GTG TGT GRC | 5 | 2 | 40 |
| 866 | CTC CTC CTC CTC CTC CTC | 1 | 1 | 100 |
| All | 13 | 52 | 34 | 65 |
